# Emphysema following air-powder abrasive treatment for peri-implantitis

**DOI:** 10.1186/s40902-018-0151-7

**Published:** 2018-05-13

**Authors:** Sung-Tak Lee, Malavika Geetha Subu, Tae-Geon Kwon

**Affiliations:** Department of Oral and Maxillofacial Surgery, School of Dentistry, Kyungpook National University, 2177 Dalgubeol-daero, Jung-gu, Daegu, 41940 Republic of Korea

**Keywords:** Subcutaneous, Pneumomediastinum, Emphysema, Peri-implantitis, Air abrasive

## Abstract

**Background:**

Subcutaneous emphysema refers to swelling caused by the presence of air or gas in the interstices of loose connective tissue. In the head and neck area, it may follow the fascial planes and is characterized by sudden swelling, crepitus on palpation, infrequent pain, and air emboli on radiography. It usually occurs as a complication in dental treatment. Some reports have described subcutaneous emphysema caused by dental procedures; however, severe emphysema related to peri-implantitis after treatment has not been documented. Accordingly, the current report describes a rare case of subcutaneous cervical emphysema resulting from the use of an air-powder abrasive device to treat peri-implantitis.

**Case presentation:**

Based on a review of the existing literature and the present case, nine cases of subcutaneous emphysema due to air-powder abrasive device have been reported. In most cases, the emphysema resolved over time after treatment with prophylactic antibiotics; among these, two were related to peri-implantitis management.

**Conclusion:**

Considering the frequent use of air-powder abrasive devices to treat peri-implantitis, the potential risk of iatrogenic emphysema related to this procedure needs to be addressed more extensively.

## Background

Subcutaneous emphysema is a potentially serious clinical complication of dental treatments involving forceful injection of air into connective tissue below the dermal layer [1]. Typical incidences are encountered during surgical extraction of teeth, restorative treatment, endodontic treatment, and the use of air-driven hand pieces [2]. The etiology of subcutaneous emphysema is related to traumatic, iatrogenic, incidental, or pathological events, all of which result from the introduction of gas between the tissue layers [3]. It has been commonly reported that air-driven dental instruments can also cause various degrees of subcutaneous or cervicofacial emphysema.

Peri-implantitis is an inflammatory process that occurs around an implant and is characterized by inflammation and loss of supporting marginal bone. Air-powder abrasive treatment is one efficient mechanical debridement method for the treatment of peri-implantitis. The abrasive powder is introduced into a stream of air to clean and polish the micro-rough implant surface by removing contaminated surfaces or smoothening the texture of the implant [4]. There is consensus in using air-polishing devices to manage peri-implantitis [4, 5]; however, the risk for development of subcutaneous emphysema has been suggested [5].

The authors encountered a very rare but severe case of cervico-facial and retropharyngeal emphysema that extended to the mediastinum as a result of using air-abrasive powder to treat peri-implantitis. The present report describes a case of subcutaneous cervical emphysema resulting from the use of an air-powder abrasive device to treat peri-implantitis. The ensuing discussion reviews the existing literature pertaining to the topic and emphasizes the possible risks of air-abrasive applications in the treatment of peri-implantitis.

## Case presentation

**Fig. 1:**
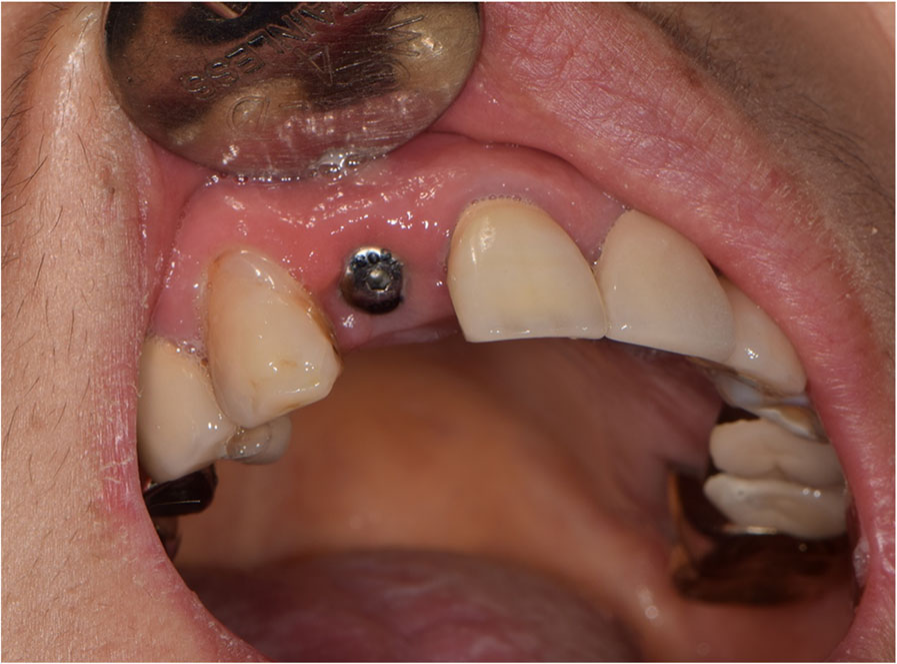
Buccal aspect of the implant (#12) treated with air-powder abrasive to manage peri-implantitis

**Fig. 2:**
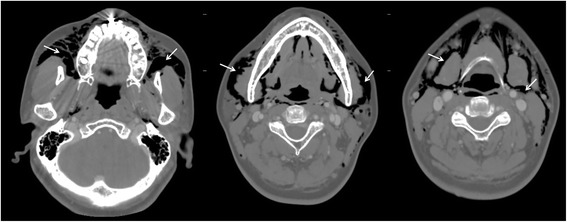
Paranasal sinus computed tomography (PNS CT [axial view]) revealing multiple air bubbles of emphysema in the buccal, infratemporal, buccal and canine spaces (arrows)

**Fig. 3:**
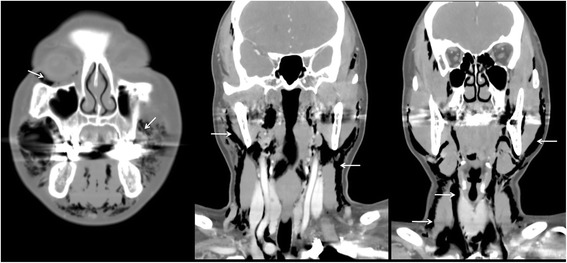
Paranasal sinus computed tomography (PNS CT [coronal view]) of emphysema in the retromandibular, visceral, and retropharyngeal spaces (arrows)

**Fig. 4:**
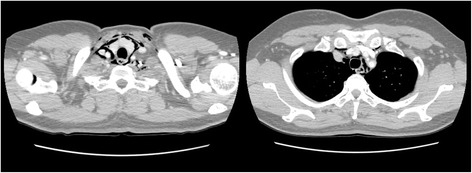
Chest computed tomography revealing emphysema extending to the mediastinum

A 51-year-old female with no remarkable medical history was referred by a private dental clinic for painful swelling, warmness, and difficulty with breathing. One hour before the referral, the patient underwent treatment for peri-implantitis on the buccal side of an implant at the right maxillary lateral incisor at a local dental clinic. To treat the peri-implantitis non-surgically, the subgingival peri-implant pocket was slightly retracted and an air-powder abrasive device was used on the exposed implant surface to clean any possible contamination. During the treatment, however, the patient began to feel facial swelling accompanied by pain, which also spread to the neck and did not disappear. The patient was immediately transferred to the authors’ institution. There were no remarkable findings near the buccal gingiva around the implant (Fig. 1). However, the patient exhibited crepitus on palpation of the right face, and both eyes were swollen and shut. Visual acuity and ocular movement were intact. The patient felt discomfort during breathing but exhibited relatively stable vital signs: body temperature 36.2 °C, oxygen (O_2_) saturation 95–96% in room air; blood pressure 150/90 mmHg; and heart rate, 88 beats/min. Chest radiography revealed pneumomediastinum, and enhanced computed tomography (CT) revealed bilateral interstitial air emphysema in the temporal, infratemporal, buccal and canine, submandibular, submental, retromandibular, retropharyngeal, visceral, and carotid spaces (Figs. 2 and 3). Severe subcutaneous emphysema in both sides of the neck extended to the mediastinum. However, abnormal fluid collection and mass lesions, or distinctive lymph node enlargement, were not observed at the lung and mediastinal region (Fig. 4). The patient also underwent consultation with the department of thoracic surgery and was subsequently admitted to the hospital to monitor lung condition and to prevent infection. The patient was advised not to increase intraoral air pressure. Prophylactic antibiotic medication (cephalosporin and piperacillin/tazobactam [intravenous]) was administered for 7 days with 100% O_2_ supply, in addition to analgesic medication for pain control. Approximately 3 days after admission, O_2_ saturation was between 98 and 100% without O_2_ supply. Ten days after admission, all facial and neck swelling virtually disappeared without any complications. Regular laboratory tests were conducted, and swings of infection were not observed; CT findings returned to normal. The patient was discharged 13 days after initial development of emphysema.

## Discussion

It has been suggested that the cause of the subcutaneous emphysema could be classified into four categories: coughing during surgery, air forced directly into the tissue, prolonged surgical procedure(s), and no specific cause [6]. If air is further pushed, it leads to inflation of facial spaces. In worst-case scenarios, the air passes through the masticator spaces into the parapharyngeal and retropharyngeal spaces and penetrates the mediastinum [7]. It has been reported that communication between the maxillofacial fascial space and mediastinum can carry a risk for bacterial spread, potentially leading to life-threatening infections of the retropharyngeal pace and the mediastinum [8].

In mild forms of emphysema, a small amount of air is forced into the tissue causing mild swelling and crepitus. If a larger amount of air is driven into the tissue, it may lead to significant pain, increased swelling, and more crepitus [9]. It has also been reported that inadvertent introduction of air into soft tissues during procedures using high-speed, air-driven hand pieces in endodontic treatments can lead to subcutaneous and mediastinal emphysema [10].

Subcutaneous emphysema is an uncommon clinical complication of dental treatment caused by the forceful injection of air into the loose surrounding connective tissue. According to a review of 75 reported cases from 1960 to 1993 [11], emphysematous complications were caused most frequently by the use of high-speed hand pieces (*n* = 42) and air syringes (*n* = 27). Only one case of implant cleaning-related emphysema was included in that case series. Centripetal air dissection with retropharyngeal and mediastinal emphysema has occurred in many patients, especially following extractions [11]. A subsequent case review (1994–2008) involving 47 cases of subcutaneous emphysema and pneumomediastinum following dental treatment reported that air-syringe comprised 15% (7 cases) of the total; however, the main cause was the use of a high-speed air turbine hand piece in 31 patients (66%) [12]. Another case review reported that 16 of 32 cases (50%) of iatrogenic subcutaneous emphysema were related to the use of air-driven hand pieces [8]. The development of pneumomediastinum after the use of air-polishing devices using abrasive powder for peri-implantitis is very rare. To our knowledge, the present report is the first to describe extensive subcutaneous emphysema accompanying pneumomediastinum after peri-implantitis treatment using an air-abrasive device.

Previous reports, including ours, addressing subcutaneous emphysema related to air-powder abrasive devices are summarized in Table 1. There have been nine case reports, among which only two cases were related to peri-implantitis treatment-related subcutaneous emphysema. Among the nine reported cases, prophylactic antibiotics were not prescribed in three [13–15]. In one case involving a 16-year-old girl, the patient’s recovery was uneventful without antibiotics, even in the presence of pneumomediastinum [14]. This difference in treatment modality may be based on severity and the patients’ general condition. In most cases, the emphysema was resorbed within 7 days.

**Table 1:** Subcutaneous emphysema caused by air-powder instruments (1987 to date)

Reference	Age, years/sex	Procedure	Suspected cause	Distribution	Treatment and antibiotics	Cx
Finlayson & Stevens (1988) [17]	49/male	Tooth stain removal	Air polishing (Cavi-jet®)	Subcutaneous	Penicillin (IV) 4 times/day for 7 days	–
Bergendal et al. (1990) [13]	40/female	Calculus removal for peri-implantitis	Air-powder abrasive (Prophy-Jet®)	Subcutaneous	Local application of 0.2% Hibitane®, no infection, no antibiotics	–
Liebenberg & Crawford (1997) [14]	16/female	Cleaning dental stains	Air-powder abrasive	Subcutaneous, pneumomediastinum	No antibiotics; resolved within 7 days	–
Strassen et al. (2011) [19]	52/male	Tartar removal	Air-powder abrasive (CaCO_2_ Powder Jet)	Subcutaneous, pneumomediastinum	Sultamicin 1.5 g 3 times/day (IV); resolved within 4 days	–
Bassetti et al. (2014) [16]	69/male^a^	Treatment for peri-implantitis	Air-powder abrasive (Air-Flow Master®, glycine powder)	Subcutaneous, pneumomediastinum	Amoxicillin/clavulinic acid 2.2 g (IV); resolved within 7 days	–
Alonso et al. (2017) [15]	73/male	Peri-implant cleaning	Air-powder polishing (sodium bicarbonate powder)	Subcutaneous	Methylprednisolone, 40 mg (intramuscular) + azithromycin, 500 mg/day for 3 days; resolved 4 days	–
	43/male	Teeth cleaning	Air-powder polishing (sodium bicarbonate powder)	Subcutaneous, pneumomediastinum	12 h of observation, no antibiotics; resolved 3 to 4 days	–
	62/female	Teeth cleaning	Air powder polishing (sodium bicarbonate powder)	Subcutaneous	Ibuprofen, 600 mg/8 h for 5 days and azithromycin 500 mg/day for 3 days; resolved within 5 days	–
Current report	51/female^a^	Cleaning for peri-implantitis	Air powder abrasive	Subcutaneous, retropharynx, pneumomediastinum	Cephalosphorin + tazobactam (IV); resolved within 10 days	–

Cavi-Jet and Prophy-Jet are registered trademarks of Dentsply Sirona, USA; Air-Flow Master is a registered trademark of EMS Dental, Nyons, France

*Cx* complications, *IV* intravenous

^a^Cases of peri-implantitis treatment-related emphysema

In the current case, it is uncertain why the emphysema spread widely and deeply into the bilateral neck and mediastinum. It has been suggested that the combination of air-polishing devices and abrasive powder may increase the velocity of abrasive particles [15]. At the same, a long epithelial lining at the peri-implant pocket with minimal bone-gingival attachment can be a factor in the development of emphysema after the use of air-abrasive powder in peri-implantitis treatment [16]. Inadvertent angulation of the nozzle tip into a gingival pocket can also lead to this condition [17]. Therefore, careful attention should be devoted to angulation and distance between the nozzle of the air-abrasive device and the implant surface, and continuous application of the devices should be discouraged.

In treating cervico-facial and retropharyngeal emphysema extending to the mediastinum, correct diagnosis is crucial. Sudden onset of neck swelling and crepitus, tenderness, erythema, edema, or respiratory disturbance establishes the diagnosis. In this patient, mild pneumothorax and pneumomediastinum were observed on posteroanterior chest X-ray and CT images. Emphysematous complications in dentistry usually have a benign course. However, extensive emphysema, similar to the case described here, requires prophylactic antibiotics, close observation of the airway, and monitoring of the progression of gas. Incision and drainage, and aggressive supportive treatment, such as chest tube placement, are sometimes necessary [18].

## Conclusions

Surgical treatment is not usually needed for subcutaneous emphysema of the cervico-facial area. However, the potential risk of iatrogenic emphysema caused by the use of air-powder abrasive devices should be recognized. Considering the frequent use of air-powder abrasive devices to treat peri-implantitis, the potential risk for iatrogenic emphysema related to this procedure needs more attention.

## Abbreviation

CT: Computed tomography
